# Association of serum reproductive hormones changes after neoadjuvant chemotherapy with hormone receptors expression alterations and survival outcomes in breast cancer

**DOI:** 10.3389/fsurg.2022.947218

**Published:** 2022-08-31

**Authors:** Ailin Lan, Yudi Jin, Yu Wang, Nan Ding, Yihua Wang, Yuran Dai, Linshan Jiang, Zhenrong Tang, Yang Peng, Shengchun Liu

**Affiliations:** Department of Breast and Thyroid Surgery, The First Affiliated Hospital of Chongqing Medical University, Chongqing, China

**Keywords:** breast cancer, reproductive hormones, neoadjuvant therapy, sex steroids, hormone receptors, progesterone

## Abstract

**Purpose:**

This study aimed to determine the effect of neoadjuvant chemotherapy (NAC) on circulating levels of reproductive hormones and evaluate the correlation of hormone changes after NAC with hormone receptors expression alterations and relapse-free survival (RFS) outcomes in breast cancer.

**Methods:**

Information from 181 breast cancer patients who received NAC was retrospectively analyzed. For hormones parameters, the median and interquartile range (IQR) were provided at baseline and the end of NAC then was compared by Wilcoxon signed-rank test. Categorical variables were represented as numbers and percentages and were compared *via* two-sided chi-square and Fisher's tests. The RFS outcomes were compared between patients according to hormone changes using the log-rank test. Univariate and multivariate survival analyses with hazard ratios (HR) and 95% confidence intervals (95% CI) were carried out using Cox regression.

**Results:**

Sex steroids including estradiol, progesterone, testosterone, and dehydroepiandrosterone sulfate (DHEAS) levels decreased significantly after NAC among both premenopausal and postmenopausal patients (all *P* < 0.05). Decreased estradiol levels were associated with reduced progesterone receptor (PR) expression (*P* = 0.030). In multivariate survival analysis, the non-decreased progesterone level was strongly associated with worse RFS (non-decreased vs. decreased, HR = 7.178, 95% CI 2.340–22.019, *P* = 0.001). Patients with decreased progesterone levels exhibited better 3-year RFS compared with those with non-decreased (87.6% vs. 58.3%, log-rank, *P* = 0.001).

**Conclusion:**

Multiple reproductive hormone levels were influenced by NAC. The change in estradiol level had a positive connection with PR expression alteration. Furthermore, an inverse association between the change in progesterone level and RFS outcomes was found. These findings may provide a theoretical basis for pre-operative endocrine therapy combined with NAC in breast cancer patients.

## Introduction

Female breast cancer, as the leading cause of global cancer incidence in 2020, has been the fifth leading cause of cancer mortality worldwide, with an estimated 2.3 million new cases and 685,000 deaths (1). Neoadjuvant chemotherapy (NAC) as a standard of care for locally advanced breast cancer, is increasingly used for all breast cancer subtypes (2, 3), aiming to reduce tumor size before surgery. However, in young patients, chemotherapy-associated ovarian damage is a major limitation of NAC (4). Several mechanisms lead to chemotherapy-induced ovarian injury and reduction of the ovarian reserve (5, 6), directly affecting endogenous sex hormone concentrations such as estradiol, progesterone, follicle-stimulating hormone (FSH), and luteinizing hormone (LH) (7). Due to the benefit seen in some studies irrespective of the hormone receptors' status, a direct cytotoxic effect of chemotherapy on the ovary and an indirect endocrine effect have been postulated (8, 9).

It is well known that reproductive hormones play a critical role in breast carcinogenesis. Available evidence has demonstrated that endogenous reproductive hormones can sustain tumor growth and are associated with breast cancer risk (10). Our previous works (11) and other studies (12, 13) found an association between pre-treatment hormones and prognosis in patients who received NAC treatment. A recent study has described the association of chemotherapy-induced ovarian failure with the improved outcome regardless of hormone receptor status (14), highlighting the vital role of ovarian function suppression after neoadjuvant/adjuvant chemotherapy in inhibiting disease progression. Although this study reveals the potential role of reproductive hormones in cancer progression, it is not clear the exact effect of hormone changes after neoadjuvant/adjuvant chemotherapy on survival outcomes. To address this question, we evaluated serum reproductive hormone changes after NAC treatment in association with survival outcomes in breast cancer patients.

Most breast malignancies are hormone-dependent, and express hormone receptors such as estrogen receptor (ER) and progesterone receptor (PR). Many studies have suggested that the expression status of ER and PR may differ between the initial diagnostic core biopsies and excisional specimens after NAC (15, 16). The changes in these receptors reveal important clinical significance as clinicians need to make appropriate treatment adjustments according to the status of these biomarkers. However, the mechanisms mediating the changes in hormone receptor expression remain unknown. Although the effect of NAC on hormone receptor expression has been widely studied, the results were controversial.

In this context, we analyzed changes in estradiol, progesterone, testosterone, FSH, LH, dehydroepiandrosterone sulfate (DHEAS), sex hormone-binding globulin (SHBG) levels, and free androgen index (FAI) after NAC treatment in pre- and postmenopausal women diagnosed with invasive breast cancer. The correlation of hormone changes after NAC with hormone receptor expression alterations and relapse-free survival (RFS) outcomes have been investigated.

## Methods

### Patients

Details of the previous retrospective study on which this research is based have been posted (11). To investigate the relationship between changes in reproductive hormone levels after NAC treatment and survival outcomes, we included people with post-treatment hormone reports from previous study populations. This retrospective study included 181 female patients with breast cancer who were administered NAC between February 2013 and December 2019 at the First Affiliated Hospital of Chongqing Medical University (Chongqing, China). The inclusion criteria were as follows: (a) all enrolled breast cancer patients accepted NAC and subsequent surgery; (b) patients with serum reproductive hormones tests before NAC start and at the end of NAC; (c) patients received standard adjuvant therapy after surgery; (d) all included patients had complete clinical records. The exclusion criteria were as follows: (a) distant metastasis before NAC; (b) synchronous bilateral breast cancer; (c) administration of oral contraceptives or other hormones in the past 6 months; (d) pregnancy or lactation in the previous 6 months; (e) prior cancer diagnosis or serious organ disease; (f) endocrine therapy, radiotherapy or targeted therapy before NAC. Details related to the demographic and pathological characteristics were collected. Meanwhile, information on the serum reproductive hormones before initial NAC and after the last cycle of NAC, including estradiol, progesterone, testosterone, FSH, LH, DHEAS, FAI, and SHBG, was gathered. A total of 91 patients were enrolled in the follow-up group between February 2013 and December 2018, and each participant was followed from the baseline exam until death or February 1, 2021. The hospitalization information system was utilized to obtain the patients' in-hospital information, and outpatient information was obtained through the hospital outpatient system, face-to-face, or by telephone.

### Measurements of serum reproductive hormones

Samples were collected at baseline and the end of NAC. Concentrations of estradiol, progesterone, testosterone, FSH, LH, DHEAS, and SHBG were determined using an electrochemiluminescence method on an immunoassay analyzer (DxI 800 Immunoassay System; Beckman Coulter, Brea, CA, United States). FAI was calculated as FAI = (testosterone/SHBG) × 100.

### Immunohistochemical staining

All mammary gland malignant tumor specimens from core biopsies and surgical excisions were reviewed by two experienced pathologists. Immunohistochemistry (IHC) was utilized to measure ER, PR, the human epidermal growth factor receptor 2 (HER2) status, and the Ki67 index before and after NAC. The Ki67 was explained as the percentage of tumor cell nuclei between 400 and 500 cells.

### Definitions

Menopause was defined as no spontaneous menses over the past 1 year or no menses for <1 year with FSH and estradiol levels in the postmenopausal range or post bilateral oophorectomy. The ER and PR status were defined positive if >1% of cancer cells were stained, and the HER2 status was defined positive if >10% of the cancer cells showed a 3+ score by IHC or a >2.2-fold change compared to the expression of chromosome enumeration probe 17 (CEP17) in cancer cells *via* fluorescence *in situ* Hybridization (FISH) (17). The hormone receptors positive was explained as ER and/or PR positive. In accordance with the expression of hormone receptors and HER2 status, the included patients were classified according to the following four subtypes: luminal (ER+ and/or PR+, HER2−), luminal/HER2 (ER+ and/or PR+, HER2+), HER2 (ER− and PR−, HER2+), and triple-negative (ER− and PR−, HER2−). Pathological complete response (pCR) was defined as no remaining invasive tumor lesions in any excised breast tissue and lymph node (ypT0/Tis ypN0) (18). The RFS was explained as the time from the baseline exam to the time of the first event (invasive local recurrence, regional recurrence, distant recurrence, invasive contralateral breast cancer, secondary malignancy, or death for any reason).

### Evaluation of neoadjuvant chemotherapy response

The response of the tumor to NAC was evaluated by ultrasound and magnetic resonance imaging. Clinical response was assessed by making a comparison of the change of the primary site. The treatment response of NAC was assessed by imaging examinations based on the Response Evaluation Criteria in Solid Tumors (RECIST) guidelines version 1.1.

### Statistical methods

For the non-normally distributed continuous parameters, the median and interquartile range (IQR) were provided at baseline and the end of NAC, then were compared by Wilcoxon signed-rank test. Categorical variables were represented as numbers and percentages and were compared *via* two-sided chi-square and Fisher's tests. The RFS outcomes were compared between patients according to hormone changes using the log-rank test. Univariate and multivariate survival analyses with hazard ratios (HR) and 95% confidence intervals (95% CI) were carried out using Cox regression. In line with the widely used recommendations (19), variables with a *P*-value lower than 0.25 in univariate analysis were selected as independent variables and analyzed by multivariate Cox regression. Cases with missing values for any of the variables in the model were excluded from the analysis. The 95% CI was utilized to express ranges within which true parameter values were likely to lie. All statistical tests were 2-sided, and *P* values less than 0.05 were considered statistically significant. Data analyses were performed using SPSS (version 26.0) software (SPSS Inc., Chicago, IL, United States).

## Results

### Population characteristics

**Figure 1:**
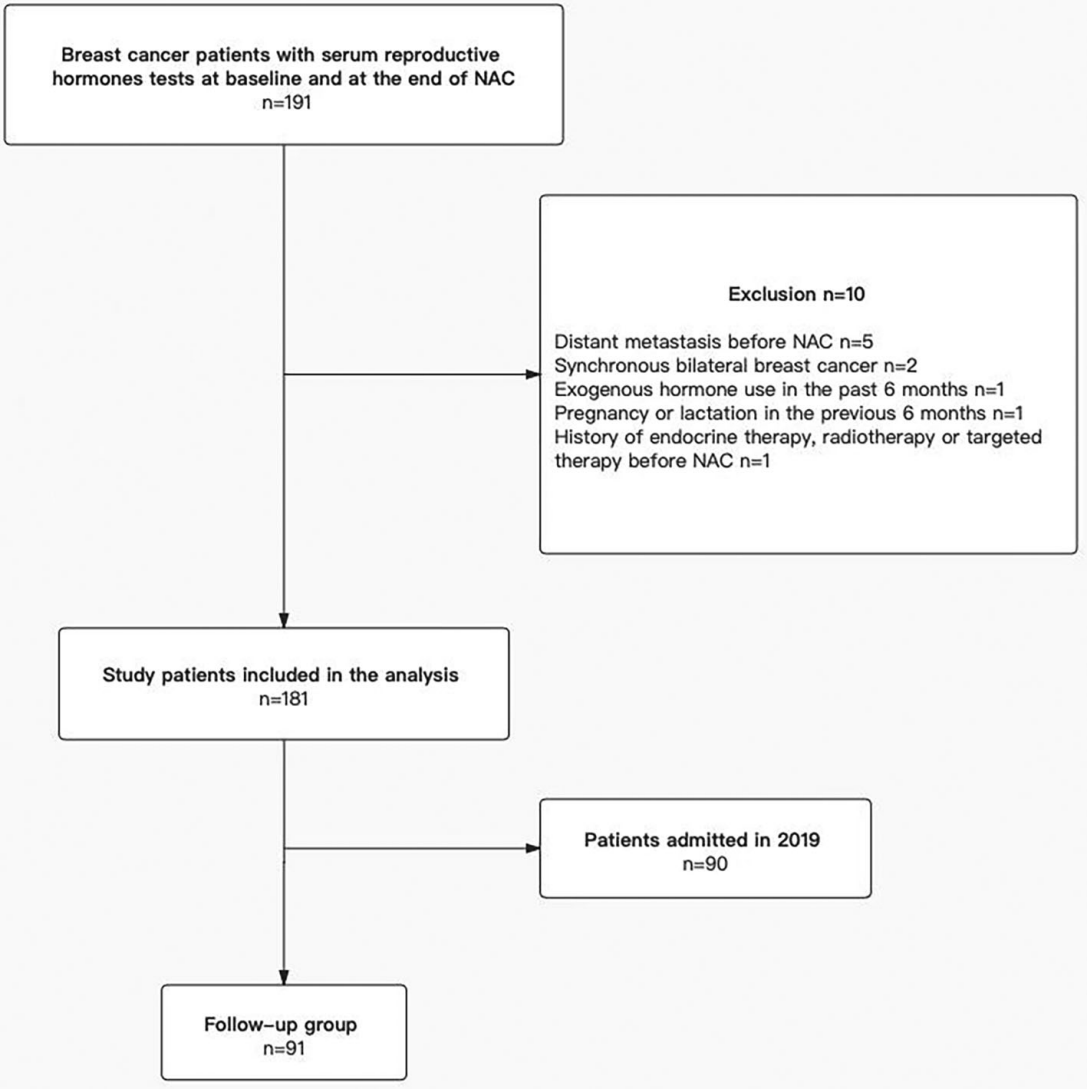
Flow chart of the patient selection. After exclusions, the final sample size was 181 patients, and a total of 91 patients were enrolled in the follow-up group. NAC, neoadjuvant chemotherapy.

The clinicopathological characteristics of the study population at enrollment are summarized in Table 1. A total of 181 patients (premenopausal group: 101; postmenopausal group: 80) with invasive breast cancer were included in the study (Figure 1). The median age of the premenopausal and the postmenopausal patients was 44 years (Range, 21–57 years) and 56 years (Range, 45–69 years) respectively. Most the premenopausal patients with a BMI < 24 kg/m^2^ (71.2%), while most of postmenopausal with a BMI ≥ 24 kg/m^2^ (62.5%). Most of premenopausal and postmenopausal patients had cT2 (70.3% vs. 72.5%) or cN1/N2 (65.3% vs. 53.8%) disease. In the IHC or FISH of the excised specimens, most of premenopausal and postmenopausal patients were ER-positive (58.4% vs. 57.5%) and HER2-negative (56.4% vs. 61.3%) and had a Ki67 index >14% (81.2% vs. 80.0%). Most patients received 4 chemotherapy cycles (85.1% vs. 78.8% in the premenopausal and the postmenopausal groups respectively). The pCR rate of the premenopausal and the postmenopausal patients was 9.9% and 8.8% respectively.

**Table 1:** Baseline clinicopathological characteristics of all patients.

Characteristics	Premenopausal (*n* = 101) *n* (%)	Postmenopausal (*n* = 80) *n* (%)
**Age (years)**		
Mean	42.4 ± 7.3	56.7 ± 5.9
Median	44	56
Range	21–57	45–69
**BMI (kg/m^2^)**		
<18.5	6 (5.9%)	3 (3.8%)
18.5–24	66 (65.3%)	27 (33.8%)
≥24	27 (26.7%)	50 (62.5%)
Not available	2 (2.0%)	0
**Tumour size**		
cT1	9 (8.9%)	9 (11.3%)
cT2	71 (70.3%)	58 (72.5%)
cT3	21 (20.8%)	13 (16.3%)
**Nodal involvement**		
cN0	16 (15.8%)	10 (12.5%)
cN1/N2	66 (65.3%)	43 (53.8%)
cN3	19 (18.8%)	27 (33.7%)
**ER status**		
Positive	59 (58.4%)	46 (57.5%)
Negative	39 (38.6%)	33 (41.3%)
Not available	3 (3.0%)	1 (1.2%)
**PR status**		
Positive	49 (48.5%)	34 (42.5%)
Negative	48 (47.5%)	45 (56.3%)
Not available	4 (4.0%)	1 (1.2%)
**HER2 status**		
Positive	40 (39.6%)	30 (37.5%)
Negative	57 (56.4%)	49 (61.3%)
Not available	4 (4.0%)	1 (1.2%)
**Molecular subtype**		
Luminal	39 (38.6%)	35 (43.8%)
Luminal/HER2	17 (16.8%)	11 (13.8%)
HER2	22 (21.8%)	18 (22.5%)
Triple negative	17 (16.8%)	15 (18.8%)
Not available	6 (5.9%)	1 (1.3%)
**Ki67 status (%)**		
≤14	15 (14.9%)	15 (18.8%)
14–30	43 (42.6%)	38 (47.5%)
>30	39 (38.6%)	26 (32.5%)
Not available	4 (4.0%)	1 (1.3%)
**Regimen**		
Anthracycline- and taxane-based	98 (97.0%)	74 (92.5%)
Anthracycline-based only	1 (1.0%)	0
Taxane-based only	2 (2.0%)	6 (7.5%)
**Chemotherapy cycles**		
<4	2 (2.0%)	4 (5.0%)
4	86 (85.1%)	63 (78.8%)
>4	13 (12.9%)	13 (16.3%)
**Responder**		
pCR	10 (9.9%)	7 (8.8%)
cCR	4 (4.0%)	3 (3.8%)
cPR	55 (54.5%)	43 (53.8%)
cSD	30 (29.7%)	26 (32.5%)
cPD	2 (2.0%)	1 (1.2%)

BMI, body mass index; ER, estrogen receptor; PR, progesterone receptor; HER2, human epidermal growth factor receptor 2; pCR, pathological complete response; cCR, clinical complete response; cPR, clinical partial response; cSD, clinical stable disease; cPD, clinical progressive disease.

### Serum reproductive hormones changes

**Figure 2:**
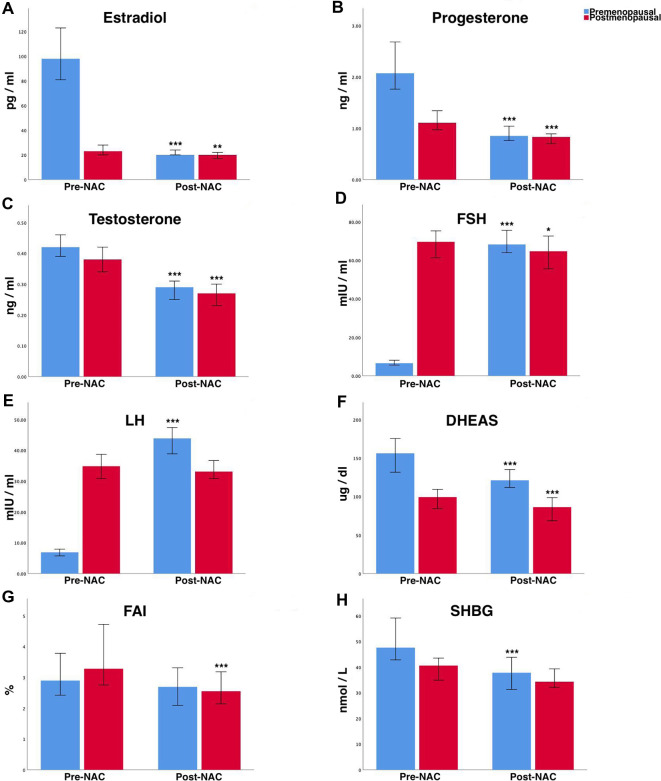
Changes of various serum reproductive hormones after NAC treatment in premenopausal and postmenopausal women with breast cancer. (**A**) Estradiol, (**B**) progesterone, (**C**) testosterone, (**D**) FSH, (**E**) LH, (**F**) DHEAS, (**G**) FAI, and (**H**) SHBG. Values are expressed as medians, and 95% CI are shown by whiskers. *P < 0.05; **P < 0.01; ***P < 0.001 (*P*-value by Wilcoxon signed-rank test). NAC, neoadjuvant chemotherapy; FSH, follicle stimulating hormone; LH, luteinizing hormone; DHEAS, dehydroepiandrosterone sulfate; FAI, free androgen index; SHBG, sex hormone-binding globulin; 95% CI, 95% confidence intervals.

Serum reproductive hormones changes after NAC in premenopausal and postmenopausal women with breast cancer as shown in Figure 2 and Table 2. In premenopausal women with breast cancer, a significant decrease in estradiol, progesterone, testosterone, DHEAS, and SHBG (all *P* < 0.001) levels was found. Meanwhile, we observed a significant increase in FSH and LH (both *P* < 0.001) levels. However, no significant changes were found in FAI (*P* > 0.05). In postmenopausal breast cancer patients, we found a significant reduction in progesterone, testosterone, DHEAS levels, and FAI (all *P* < 0.001). Concurrently, a slightly significant decrease in estradiol and FSH (both *P* < 0.05) levels was observed. No significant changes were found in LH and SHBG (both *P* > 0.05) levels.

**Table 2:** Serum reproductive hormones changes after NAC treatment in premenopausal and postmenopausal women with breast cancer.

Factors	Premenopausal (*n* = 101)			Postmenopausal (*n* = 80)		
	Pre-NAC Median (IQR)	Post-NAC Median (IQR)	*P* value^a^	Pre-NAC Median (IQR)	Post-NAC Median (IQR)	*P* value^a^
Estradiol (pg/ml)	98 (57–172)	20 (13–40)	<0.001	23 (16–34)	20 (12–29)	0.007
Progesterone (ng/ml)	2.07 (1.33–9.43)	0.85 (0.61–1.28)	<0.001	1.11 (0.88–1.60)	0.83 (0.50–1.05)	<0.001
Testosterone (ng/ml)	0.42 (0.33–0.56)	0.29 (0.17–0.36)	<0.001	0.38 (0.25–0.51)	0.27 (0.15–0.38)	<0.001
FSH (mIU/ml)	6.53 (4.81–15.47)	68.19 (55.57–87.47)	<0.001	69.54 (50.91–89.26)	64.64 (51.08–83.71)	0.013
LH (mIU/ml)	6.90 (3.77–15.36)	43.88 (28.86–56.51)	<0.001	34.85 (23.90–45.39)	33.11 (21.16–47.16)	0.093
DHEAS (ug/dl)	156.1 (110.9–205.4)	121.0 (82.5–163.0)	<0.001	99.3 (57.9–135.5)	86.2 (50.0–117.9)	<0.001
FAI %	2.90 (1.66–4.86)	2.69 (1.29–4.16)	0.283	3.28 (2.02–5.59)	2.55 (1.50–4.14)	<0.001
SHBG (nmol/l)	47.6 (37.6–74.3)	37.8 (22.7–60.0)	<0.001	40.6 (27.7–49.7)	34.3 (27.5–48.1)	0.112

NAC, neoadjuvant chemotherapy; IQR, interquartile range; FSH, follicle stimulating hormone; LH, luteinizing hormone; DHEAS, dehydroepiandrosterone sulfate; FAI, free androgen index; SHBG, sex hormone-binding globulin.

^a^ *P*-value by Wilcoxon signed-rank test.

### Association between serum reproductive hormones changes and alterations of hormone receptors expression

Table 3 summarized reproductive hormone changes with alterations in hormone receptors expression. Decreased estradiol levels associated with reduced PR expression were found (*P* = 0.030). Similar results were found among patients with hormone receptors positive (Table 4), decreased testosterone (*P* = 0.043), as well as estradiol (*P* = 0.042) levels, were associated with reduced PR expression. However, no significant connections between various reproductive hormone changes and alterations of ER expression were observed in all patients as well as patients with hormone receptors positive (all *P* > 0.05).

**Table 3:** Analysis of reproductive hormone changes associated with alterations of hormone receptors expression in all patients.

Factors		Expression of ER					Expression of PR				
		Decrease		No decrease		*P* value^a^	Decrease		No decrease		*P* value^a^
		*n*	Valid %	*n*	Valid %		*n*	Valid %	*n*	Valid %	
Estradiol	Decrease	22	18.8	95	81.2	0.189	38	34.9	71	65.1	0.030
	No decrease	8	30.8	18	69.2		3	12.0	22	88.0	
Progesterone	Decrease	25	20.8	95	79.2	1.000	35	31.8	75	68.2	0.628
	No decrease	5	21.7	18	78.3		6	25.0	18	75.0	
Testosterone	Decrease	25	21.9	89	78.1	0.553	35	33.0	71	67.0	0.118
	No decrease	3	13.6	19	86.4		3	14.3	18	85.7	
FSH	Decrease	7	16.7	35	83.3	0.503	10	25.0	30	75.0	0.417
	Increase	23	22.8	78	77.2		31	33.0	63	67.0	
LH	Decrease	15	30.0	35	70.0	0.057	13	26.5	36	73.5	0.560
	Increase	15	16.1	78	83.9		28	32.9	57	67.1	
DHEAS	Decrease	15	16.9	74	83.1	0.308	26	32.1	55	67.9	0.383
	Increase	9	26.5	25	73.5		8	23.5	26	76.5	
FAI	Decrease	17	20.7	65	79.3	0.810	24	31.6	52	68.4	0.528
	Increase	7	17.1	34	82.9		10	25.6	29	74.4	
SHBG	Decrease	18	20.7	69	79.3	0.632	23	28.4	58	71.6	0.823
	Increase	6	16.7	30	83.3		11	32.4	23	67.6	

ER, estrogen receptor; PR, progesterone receptor; FSH, follicle stimulating hormone; LH, luteinizing hormone; DHEAS, dehydroepiandrosterone sulfate; FAI, free androgen index; SHBG, sex hormone-binding globulin.

^a^ *P*-value by two-sided chi-square and Fisher's tests.

**Table 4:** Analysis of reproductive hormone changes associated with alterations of hormone receptors expression in patients with hormone receptors positive.

Factors		Expression of ER					Expression of PR				
		Decrease		No decrease		*P* value^a^	Decrease		No decrease		*P* value^a^
		*n*	Valid %	*n*	Valid %		*n*	Valid %	*n*	Valid %	
Estradiol	Decrease	22	29.7	52	70.3	0.094	38	52.8	34	47.2	0.042
	No decrease	8	57.1	6	42.9		3	21.4	11	78.6	
Progesterone	Decrease	25	33.3	50	66.7	0.966	35	48.6	37	51.4	0.775
	No decrease	5	38.5	8	61.5		6	42.9	8	57.1	
Testosterone	Decrease	25	36.2	44	63.8	0.448	35	52.2	32	47.8	0.043
	No decrease	3	21.4	11	78.6		3	21.4	11	78.6	
FSH	Decrease	7	26.9	19	73.1	0.462	10	38.5	16	61.5	0.348
	Increase	23	37.1	39	62.9		31	51.7	29	48.3	
LH	Decrease	15	46.9	17	53.1	0.065	13	40.6	19	59.4	0.375
	Increase	15	26.8	41	73.2		28	51.9	26	48.1	
DHEAS	Decrease	15	28.8	37	71.2	0.416	26	51.0	25	49.0	0.311
	Increase	9	40.9	13	59.1		8	36.4	14	63.6	
FAI	Decrease	17	34.0	33	66.0	0.793	24	49.0	25	51.0	0.623
	Increase	7	29.2	17	70.8		10	41.7	14	58.3	
SHBG	Decrease	18	35.3	33	64.7	0.593	23	45.1	28	54.9	0.800
	Increase	6	26.1	17	73.9		11	50.0	11	50.0	

ER, estrogen receptor; PR, progesterone receptor; FSH, follicle stimulating hormone; LH, luteinizing hormone; DHEAS, dehydroepiandrosterone sulfate; FAI, free androgen index; SHBG, sex hormone-binding globulin.

^a^ *P*-value by two-sided chi-square and Fisher's tests.

### RFS outcomes and the association with serum reproductive hormones changes

**Figure 3:**
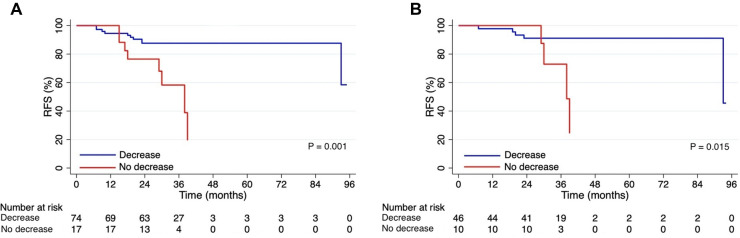
Kaplan-Meier curves display RFS by changes in progesterone levels. (**A**) Survival curves for the follow-up subgroup of 91 patients. (**B**) Survival curves for 56 patients with hormone receptors positive. RFS, relapse-free survival.

As of Feb. 1, 2021, we conducted follow-ups among a total of 91 patients (the loss rate of follow-up was 4.4%), and the clinicopathological characteristics of the study population at baseline were summarized in Table 5. The median follow-up time was 33 months (95%CI 31.3–34.7). Among the retrieved variations of reproductive hormone parameters of all patients in the follow-up subgroup, changes in estradiol (*P* = 0.022) and progesterone (*P* = 0.001) levels were associated with RFS outcomes (Table 6). These factors were then included in the Cox regression and the results were displayed in Table 7. On univariate analysis the following factors associated with worse RFS outcomes were found: younger age, premenopausal, lower BMI, larger clinical tumor size, hormone receptors negative, higher Ki67 index, decreased estradiol levels, and non-decreased progesterone levels (all *P* < 0.25). On multivariate analysis, the change of progesterone level (no decrease vs. decrease, HR = 7.178, 95% CI 2.340–22.019, *P* = 0.001) was an independent significant factor of RFS outcomes rather than the change of estradiol level. Comparison of the 3-year RFS between the decreased and the non-decreased progesterone levels groups were 87.6% and 58.3% respectively (log-rank, *P* = 0.001; Figure 3A). Similar trends were found among patients with hormone receptors positive, the survival analysis plotted as Figure 3B showed that patients with decreased progesterone levels exhibited better 3-year RFS compared with those with non-decreased (91.1% vs. 72.9%, log-rank, *P* = 0.015).

**Table 5:** Baseline clinicopathological characteristics of 91 patients in the follow-up subgroup.

Characteristics	Number of cases (%)
**Age (years)**	
≤50	61 (67.0%)
>50	30 (33.0%)
**Menstrual status**	
Premenopausal	60 (65.9%)
Postmenopausal	31 (34.1%)
**BMI (kg/m^2^)**	
<18.5	5 (5.5%)
18.5–24	45 (49.5%)
≥24	39 (42.9%)
Not available	2 (2.2%)
**Tumour size**	
cT1	12 (13.2%)
cT2	65 (71.4%)
cT3	14 (15.4%)
**Nodal involvement**	
cN0	21 (23.0%)
cN1/N2	61 (67.0%)
cN3	9 (9.9%)
**ER status**	
Positive	56 (61.5%)
Negative	34 (37.4%)
Not available	1 (1.1%)
**PR status**	
Positive	41 (45.1%)
Negative	48 (52.7%)
Not available	2 (2.2%)
**HER2 status**	
Positive	33 (36.3%)
Negative	58 (63.7%)
**Molecular subtype**	
Luminal	41 (45.1%)
Luminal/HER2	15 (16.5%)
HER2	18 (19.8%)
Triple negative	16 (17.6%)
Not available	1 (1.1%)
**Ki67 status (%)**	
≤14	16 (17.6%)
14–30	42 (46.2%)
>30	31 (34.1%)
Not available	2 (2.2%)
**Regimen**	
Anthracycline- and taxane-based	90 (98.9%)
Anthracycline-based only	1 (1.1%)
Taxane-based only	0
**Chemotherapy cycles**	
<4	1 (1.1%)
4	87 (95.6%)
>4	3 (3.3%)
**Responder**	
pCR	8 (8.8%)
cCR	5 (5.5%)
cPR	47 (51.6%)
cSD	28 (30.8%)
cPD	3 (3.3%)

BMI, body mass index; ER, estrogen receptor; PR, progesterone receptor; HER2, human epidermal growth factor receptor 2; pCR, pathological complete response; cCR, clinical complete response; cPR, clinical partial response; cSD, clinical stable disease; cPD, clinical progressive disease.

**Table 6:** Kaplan–meier analysis of RFS by reproductive hormones changes.

Factors	RFS *P*^a^
Estradiol (decrease vs. no decrease)	0.022
Progesterone (decrease vs. no decrease)	0.001
Testosterone (decrease vs. no decrease)	0.801
FSH (decrease vs. increase)	0.335
LH (decrease vs. increase)	0.432
DHEAS (decrease vs. increase)	0.976
FAI (decrease vs. increase)	0.186
SHBG (decrease vs. increase)	0.387

RFS, recurrence-free survival; FSH, follicle-stimulating hormone; LH, luteinizing hormone; DHEAS, dehydroepiandrosterone sulfate; FAI, free androgen index; SHBG, sex hormone-binding globulin.

^a^ *P*-value by log-rank test.

**Table 7:** Univariate and multivariate analysis for RFS in the follow-up subgroup of 91 patients.

Factors	Univariate analysis			Multivariate analysis		
	HR	95% CI	*P* value	HR	95% CI	*P* value
Age (year)	0.950	0.898–1.006	0.078	0.988	0.900–1.085	0.800
Menopausal status (pre vs. post)	1.992	0.643–6.170	0.232	1.305	0.248–6.863	0.753
BMI (kg/m^2^)	0.839	0.714–0.986	0.033	0.931	0.774–1.119	0.446
Clinical tumor size (cm)	1.269	1.000–1.610	0.050	1.118	0.859–1.455	0.405
Clinical axillary lymph node: pos vs. neg	1.378	0.439–4.330	0.583	–	–	–
Hormone receptors status: pos vs. neg	0.512	0.203–1.293	0.157	0.590	0.173–2.005	0.398
HER2 status: pos vs. neg	0.893	0.335–2.382	0.822	–	–	–
Ki67	1.014	0.994–1.035	0.163	1.011	0.980–1.044	0.483
Chemotherapy cycle	0.762	0.267–2.179	0.612	–	–	–
pCR: no vs. yes	1.510	0.200–11.393	0.690	–	–	–
Change of estradiol level: no decrease vs. decrease	0.032	0.000–3.960	0.162	<0.001	0.000– (1.526 × 10^192^)	0.955
Change of progesterone level: no decrease vs. decrease	4.559	1.747–11.898	0.002	7.178	2.340–22.019	0.001

Age, BMI, clinical tumor size, Ki67 index and chemotherapy cycle were entered into the model as continuous variables.

RFS, recurrence-free survival; HR, hazard ratio; CI, confidence interval; BMI, body mass index; HER2, human epidermal growth factor receptor 2; pCR, pathological complete response.

## Discussion

The main finding of this study was that reproductive hormone levels were influenced by NAC treatment, having connections with hormone receptor expression alterations and RFS outcomes in women diagnosed with invasive breast cancer. To our knowledge, this is the first study to explore the correlation of serum reproductive hormone changes after NAC with hormone receptor expression alterations and survival outcomes in breast cancer. Though our previous study revealed that pre-treatment hormones have a predicted effect (11), the dynamics of reproductive hormones may be a better predictor of survival than pre-treatment hormones.

The changes in estradiol, FSH, and LH levels in premenopausal patients observed in our study are consistent with published studies reporting that the majority of young women receiving (neo)adjuvant chemotherapy rendered postmenopausal after chemotherapy (8, 14), although these studies focus on menstrual status or ovarian function rather than hormones. In postmenopausal patients, a slightly significant decrease in FSH levels and no significant changes in LH levels were observed in our study, as previously reported (7). In addition, we found that sex steroids including estradiol, progesterone, testosterone, and DHEAS levels decreased significantly after NAC among both premenopausal and postmenopausal patients. These changes in the hormonal profile of pre- and postmenopausal patients may be due to the ovarian and adrenal dysfunction produced by the chemotherapy treatment.

In our study, nearly all patients received anthracycline–taxane combination chemotherapy. The impact of chemotherapeutic agents on reproductive hormones is different due to the mechanism of action of anticancer drugs being diverse. The mechanism by which NAC, particularly alkylating agents, which show the highest level of gonadotoxicity, contributes to ovarian damage is unclear, but it could be related to apoptotic oocyte death in primordial follicles entering the differentiation stage, which is especially susceptible to chemotherapeutic drugs effects (20, 21). Thus, cyclophosphamide can cause ovarian function impairment and leads to a decrease in sex steroids. Doxorubicin can destroy the endothelium of blood vessels and lead to a reduction in ovarian blood flow. Taxane can disrupt normal polymerization/depolymerization, causing cell cycle arrest and apoptosis (22). The previous meta-analysis indicated that the incidence of chemotherapy-induced amenorrhea was lower in anthracycline-based compared to anthracycline–taxane combination chemotherapy (23), showing that the combination of anthracycline and taxane has a greater effect on hormones.

Even if there is no report that a cytotoxic chemotherapeutic agent induces adrenal dysfunction, the not rare incidence of adrenal insufficiency in advanced gastric cancer patients who received chemotherapy was reported in a prospective study (24).

The two major sites of *de novo* steroid hormone biosynthesis in a woman are the ovaries and adrenals, and both these organs produce active estrogens, progestogens, and androgens (25). The major sex steroids produced by the ovaries are estrogens and progestogens, while the adrenals are androgens. The loss of adrenal function in the presence of intact ovaries has been shown to result in a > 90% loss of androgen production (26), highlighting the vital role of adrenals in androgen synthesis. It is well known that the production of estrogen by the ovary decreases drastically after menopause. Although it was initially thought that estrogen production ceased completely, it is now clear that postmenopausal ovaries continue to produce estrogen, albeit at significantly reduced levels (27). Other sites of estrogen synthesis become increasingly important in the postmenopausal state. Extragonadal sites such as adrenals become the main source of sex steroids during menopause (25). Thus, chemotherapy-induced ovarian and adrenal dysfunction may result in a decrease in estrogens, progestogens, and androgens in both pre- and postmenopausal patients.

Alterations of hormone receptor status in breast cancer after NAC treatment have been described in published studies (15, 16). Whether these changes are due to NAC treatment, hormones, tumor heterogeneity, sampling or technical issues need to be further clarified. We observed that decreased estradiol levels were associated with reduced PR expression, which was supported by several studies that indicated that PR decrease was more obvious in premenopausal patients (28, 29). Furthermore, PR was also found to have the highest conversion rates in NAC and adjuvant chemotherapy (30, 31). Previous studies showed that pre-operative endocrine therapy, pre-operative chemoendocrine therapy, and NAC induced a significant decrease in the expression of PR in breast cancer patients with hormone receptors positive (32, 33), highlighting the potential role of sex hormones in PR expression, which is consistent with our finding that decreased estradiol and testosterone levels were associated with reduced PR expression in patients with hormone receptors positive. Possible mechanisms involve the survival mechanism of malignant cells by changing the specific molecular pathways following the changes in hormone levels resulting in resistance to a specific treatment (28).

PR plays an important role in normal breast development and has been linked to breast cancer. Moreover, PR has generally been considered solely as an indicator of ER functionality and responsiveness to endocrine therapy (34). The expression pattern of the hormone receptors such as ER and PR has been used clinically to guide therapy and predict survival outcomes. However, discordant results have been reported regarding whether a changed receptor status will affect the predictive or prognostic parameters. Although some studies suggested that PR loss may reflect a relatively poor response to chemotherapy and may be associated with a poor prognosis (35, 36), no consensus has been drawn on this topic.

Progesterone and its receptor are increasingly gaining attention for their emerging role as critical regulators of breast malignancies. Their complex and tightly regulated actions have been challenging to determine. The role of endogenous progesterone in breast malignancy pathogenesis remains largely unexplored to date, and the proliferative/antiproliferative effects of progestins continue to be debated. Inconsistent evidence showed that progesterone can facilitate, restrain, or have no action on the invasion and migration of breast cancer cells (25).

Epidemiologic studies evaluating circulating levels of progesterone remain limited. Previous clinical studies reporting increased breast cancer risk with the use of contraceptives or menopausal hormone therapy have primarily evaluated exogenous synthetic progestogen (progestin) use, rather than endogenous progesterone (37). The potential role of endogenous progesterone in breast cancer cannot be inferred from these studies.

In what appears to be the only prior study of progesterone and survival outcomes published to date, pre-operative progesterone treatment resulted in greater than 10% absolute improvement in 5-year disease-free survival among node-positive breast cancer patients independent of their PR status (38). In contrast to this study, patients with decreased progesterone levels exhibited better 3-year RFS compared with those with non-decreased in our study. The explanation could be that non-decreased progesterone concentrations after NAC may reflect a relatively poor response to chemotherapy or resistance to later endocrine therapy. These inconsistent results could be mainly attributable to different sources of progesterone, considering that the previous study evaluated progesterone in an exogenous state while our study evaluated the circulating endogenous progesterone levels in a neoadjuvant setting. In addition, relative concentrations of progesterone metabolites may be the potential factors contributing to these discordant results. With the proliferative/antiproliferative effects of different progesterone metabolites in breast cancer having been gradually explored, the importance of progesterone metabolites in breast cancer is highlighted. However, much of the difficulty lies in defining which progesterone and its metabolites exert their effects on different pathological types of breast cancer. Limited available *in vivo* evidence suggested the effects of progesterone metabolites on breast physiology and carcinogenesis (39). A previous study reported that the cancer-promoting effects of administered progesterone were in fact due to the locally produced progesterone metabolite, but not progesterone itself (40).

Progesterone also works in hormone receptor-negative breast malignancies. A recent study reported that progesterone accelerated cell growth *in vitro* and tumorigenesis *in vivo* in a triple-negative model (41), which supported our view.

The present study undoubtedly has limitations. A single blood sample may provide an inexact measure of long-term average hormone levels; however, a separate reproducibility study manifested moderate to high stability over 2 years in average intraclass correlation coefficients of all hormones measured (39). Serum reproductive hormone levels wave motion during the menstrual cycle in premenopausal women. In addition, data on postoperative adjuvant therapy was not available, but all treatment options are recommended in accordance with current guidelines. All included HER2-positive patients did not receive pre-operative anti-HER2 targeted therapy due to financial and medical insurance reasons, hence the pCR rates of this cohort were lower than those reported in other literature (2). The major limitation of the study is that it was a retrospective study in one single center, with a not large sample size and a shorter follow-up time (most patients were included between January 2017 and December 2019); thus, it was also partly limited by selection bias. Because of the above limitations, we only conducted a preliminary correlation study between RFS outcomes as well as alterations of hormone receptors expression and reproductive hormones changes after NAC. Therefore, it is necessary to perform long-term clinical application of a large sample to make a more objective analysis and support our results.

## Conclusion

In conclusion, our results showed that sex steroids (including estradiol, progesterone, testosterone, and DHEAS) are affected by NAC and their levels are significantly reduced among both premenopausal and postmenopausal patients. Decreased estradiol levels were associated with reduced PR expression. More importantly, patients with decreased progesterone levels after NAC exhibited better 3-year RFS. Reproductive hormone levels should be part of the routine workup for patients with newly diagnosed breast cancer.

## Data Availability

The raw data supporting the conclusions of this article will be made available by the authors, without undue reservation.
